# Reverse Transcription Can Critically Impact the Diagnostic Outcome of *BCR::ABL1* Quantitative Real-Time RT-PCR

**DOI:** 10.3390/cancers15153914

**Published:** 2023-08-01

**Authors:** Birgit Spiess, Helga Kleiner, Irina Tarnopolscaia, Nicole Naumann, Alice Fabarius, Wolf-Karsten Hofmann, Susanne Saussele, Wolfgang Seifarth

**Affiliations:** Department of Hematology and Oncology, Medical Faculty Mannheim, Heidelberg University, 68167 Mannheim, Germany; helga.kleiner@medma.uni-heidelberg.de (H.K.); irina.tarnopolskaia@medma.uni-heidelberg.de (I.T.); nicole.naumann@medma.uni-heidelberg.de (N.N.); alice.fabarius@medma.uni-heidelberg.de (A.F.); w.k.hofmann@medma.uni-heidelberg.de (W.-K.H.); susanne.saussele@medma.uni-heidelberg.de (S.S.); wolfgang.seifarth@medma.uni-heidelberg.de (W.S.)

**Keywords:** chronic myeloid leukemia (CML), *BCR::ABL1* monitoring, minimal residual disease, cDNA synthesis, reverse transcriptase, specific priming, random hexamer priming, strand displacement synthesis activity

## Abstract

**Simple Summary:**

Reverse transcriptases (RT) play a crucial role in *BCR::ABL1* fusion transcript monitoring of chronic myeloid leukemia (CML). RT enzyme and reaction conditions may contribute to impairment of stoichiometry in cDNA synthesis potentially biasing in qRT-PCR data. We have comparatively investigated the performance of the MLV-RT and SuperScript IV with random-hexamer vs. target-specific priming by means of the Acrometrix™ *BCR::ABL1* reference panel and 37 clinical specimens. Our experiments identified priming type and RT type as major factors for diagnostic data variation. Variation was mainly due to different efficacies of RT enzymes to process low- (<50) and high-copy targets. The impairment of the high-performing SuperScript IV in processing low- (*BCR::ABL1*) and high-copy-number (*GUSB* or *ABL1*) RNA targets equally was not reflected by the diagnostically relevant Log (*BCR::ABL1/GUSB*%) values. For improving *BCR::ABL1* assay sensitivity with a faithful representation of diagnostic targets, increased RNA/cDNA amounts and distinct RT/priming combinations are highly recommended.

**Abstract:**

Reverse transcriptases (RT) are essential tools in *BCR::ABL1* fusion transcript monitoring in chronic myeloid leukemia (CML). The RT type and cDNA priming method may impair the stoichiometry of cDNA synthesis, thereby potentially introducing a bias in *BCR::ABL1* qRT-PCR data. Using the Acrometrix™ *BCR::ABL1* reference panel and 37 clinical specimens, we have comparatively investigated the performance of the RTs MLV and SuperScript IV with random hexamer vs. target-specific priming. Quantitative RT-PCR results identified the priming type and RT type as major factors for diagnostic data variation, mainly due to the different efficacies of processing *BCR::ABL1* low-copy-numbers (<50) compared to *GUSB* or *ABL1* high-copy targets. The impairment of SuperScript IV in processing low- and high-copy-number RNA targets equally was not reflected by the diagnostically relevant Log (*BCR::ABL1/GUSB*%) values. Therefore, the correct representation of housekeeping and *BCR::ABL1* target genes should have priority when aiming at as high a number of housekeeping gene copies as possible. Our data suggest that for improving *BCR::ABL1* assay sensitivity, increased RNA/cDNA amounts and the use of distinct RT/priming combinations are advantageous. However, for inter-laboratory harmonization, the proper conversion factor according to the CML international standard (IS) has to be reevaluated each time the grade of RT is changed.

## 1. Introduction

### 1.1. Chronic Myeloid Leukemia (CML) and Assessment of Molecular Response (MR)

In the molecular diagnosis of CML and the monitoring of the molecular response to tyrosine kinase inhibitor (TKI) treatment, qRT-PCR is considered the gold standard [1,2]. Milestones defined by the ELN according to Log reduction have been used to define the optimal response or treatment failure. Moreover, deep molecular remission (DMR), defined as *BCR::ABL1* < 0.01% IS lasting for at least 2 years, is crucial for the decision of TKI treatment discontinuation. A strict technical standardization of *BCR::ABL1* transcript measurements is mandatory, which also includes the selection of the most suitable control genes [1,3,4,5,6,7]. Employing *ABL1* or *GUSB* as control genes, current state-of-the-art qRT-PCR techniques are able to detect up to a 5-Log reduction in *BCR::ABL1*. The CML Working Group within the European Leukemia Net (ELN) has established definitions of MR that also reflect the sensitivity of the respective molecular tests used. MR4 indicates 4-Log reduction (*BCR::ABL1* IS 0.01%), MR^4.5^ indicates 4.5-Log reduction (*BCR::ABL1* IS 0.0032%), and MR^5^ indicates 5-Log reduction (*BCR::ABL1* international standard (IS) 0.001%). There, the absolute copy numbers of house-keeping genes serve as indicators of RNA quality and grant the highest degree of qRT-PCR fidelity, accuracy, and reproducibility [5,8,9]. Since all pre-PCR steps, starting from total RNA extraction to the quantitative co-amplification of *BCR::ABL1* and an internal housekeeping gene (*GUSB* or *ABL1*), can significantly affect the results, all steps must be continuously checked by an adequate quality and validation management [8,10,11,12]. A comprehensive analysis of factors contributing to cDNA synthesis efficacy was recently performed by Jeromin and co-workers, who found that the use of high-fidelity RT such as SuperScript IV (ThermoFisher Scientific, Waltham, MA, USA) can push the sensitivity of *BCR::ABL1* monitoring to a higher level [13].

### 1.2. Polymerization Activities of RTs and Potential Impact on BCR::ABL1 Monitoring 

Reverse transcriptases are the most relevant tools in gene expression analysis and life science research [14]. Under native conditions, retroviral RTs are able to proceed through relatively long stretches of RNA/DNA and DNA/DNA duplices [15,16]. Under native conditions, the strand displacement (SD) synthesis activity of RTs is necessary in vivo for the completion of the reverse transcription of retroviral RNA genomes, as demonstrated for MLV [17,18]. 

As widely acknowledged, in CML, an appropriate measure of molecular responses defined as *BCR::ABL1*/housekeeping gene (HKG) % levels on the IS is required. Therefore, it is preferable that the RT enzymes perform a stoichiometrically correct reverse transcription, i.e., for each RNA molecule, just one cDNA molecule is generated. Particularly, low- (*BCR::ABL1*) and high-copy-number (*GUSB* or *ABL1*) targets should be proportionally processed with identical efficacy within a defined range of sample RNA concentrations. However, there are numerous reports on how the yield of cDNA from clinical samples could be improved, thereby challenging the question regarding the desired 1:1 stoichiometry [11,13,19,20,21,22]. In fact, it seems technically difficult to robustly judge different RTs and distinguish between errors found in the RNA preparations used as a template and those caused by the RT itself or the type of priming (specific, random, oligo-dT, or a mixture of the latter) [23]. 

Aiming at the improvement of diagnostic sensitivity in CML monitoring, we set out to comparatively investigate the impact of two different RTs (MLV RT vs. SuperScript IV RT) and the type of priming (random vs. specific priming) on *BCR::ABL1* assay performance. After subsequent highly standardized TaqMan qPCR on the Acrometrix™ *BCR::ABL1* panel and clinical specimens (*n* = 37), we report the resulting Log levels (*BCR::ABL1*/HKG%) and discuss the best possible authentic measurements of the *BCR::ABL1* load by choosing the optimal combination of the RT enzyme and cDNA priming. 

## 2. Materials and Methods

### 2.1. Clinical Samples, Controls, and Ethical Considerations

As one of the German national reference laboratories for CML diagnostics, the strict international guidelines for CML monitoring were applied in our study [5,9,24]. Total RNA was extracted from clinical samples, employing the automated Maxwell1MDx technology (Promega, Mannheim, Germany). Peripheral blood (PB) samples of healthy volunteers were included in all processes to serve as a negative control for spillover and/or cross-contaminations during the RNA extraction and cDNA synthesis steps. In case of the false positivity of the control samples, all steps were repeated, starting from frozen backup material. Control blood samples of healthy donors were derived, fully anonymized, from the local blood bank. Written informed consent was obtained in accordance with the declaration of Helsinki for all human specimens used in this study. The diagnostic analyses performed according to Good Clinical Practice (GCP) guidelines included a total of 37 CML patients from various clinical trials (Dasfree, 2012-001421-27; DasaHIT, 2015-003502-16; ENDURE CML, 2016-001030-94) and were approved by the local Ethics Committee (Medizinische Ethikkommision II der Medizinischen Fakultät Mannheim der Ruprecht Karls-Universität Heidelberg, 2013-509N-MA and 212-247-AWB-MA). In general, RNA leftovers or frozen backup material from routine testing was used for our investigation. In accordance with the declaration of Helsinki, written informed consent was obtained from all patients (Table 1). 

### 2.2. The AcroMetrix™ BCR::ABL1 Reference Panel

To simulate the different Log levels of *BCR::ABL1* monitoring (% *BCR::ABL1*/*ABL1* value (IS)) by qRT-PCR analysis, the AcroMetrix™ BCR-ABL Panel (ThermoFisher Scientific), the first commercially available cell-based secondary reference panel for *BCR::ABL1* quantification on the IS [25], was used. The panel includes five vials, each containing a distinct lyophilized mixture of the human cell lines K562 (*BCR::ABL1* e14a2 fusion gene positive) and HL60 cells (*BCR::ABL1* negative). Each vial contains 1 × 10^6^ cells, in total. Overall, the AcroMetrix™ panel comprises panels A (10% BCR-ABL/ABL), B (1% *BCR::ABL1*/*ABL1*), C (0.1% *BCR::ABL1*/*ABL1*), D (0.01% *BCR::ABL1*/*ABL1*), and E (0.0032% *BCR::ABL1*/*ABL1*) and is intended for being used as an external control panel for the analytical evaluation of *BCR::ABL1* test methods. We have employed the AcroMetrix™ *BCR::ABL1* reference panel (lot no. 043019, ref. no. 956980) for investigating the potential impact of different RT and priming combinations on diagnostic *BCR::ABL1* monitoring data. The resuspendation of lyophilisate and the extraction of RNA were performed according to the manual of the manufacturer.

### 2.3. cDNA Synthesis Employing Random Hexamer and Single Specific Priming Using MLV Reverse Transcriptase

One commercially available RT/cDNA first-strand synthesis kit used was the MLV reverse transcriptase system (200 U/µL) (Invitrogen/Thermofisher Scientific, Waltham, MA, USA, cat. no. 28025013). This RT is also being used in our routine CML monitoring diagnostics. For random cDNA synthesis, 1 µg of total RNA was diluted in 19 µL of bidest. H_2_O. The solution was incubated at 65 °C for 10 min and then 3 min on ice. Consecutively, 21 µL of RT-Mix was added, containing 12 U/µL MLV RT, 1.2 U RNasin Plus (Promega), dNTPs (10 mM each), 2 mM DTT, 2 × first strand buffer, and 0.1 U A260 (400 ng = ~200 pg pmol pdN6 random primer (Roche, Mannheim). The solution was incubated for 2 h at 37 °C, followed by 5 min at 65 °C, and then stored at −20 °C. For the specific priming of cDNA synthesis, 1 µg of total RNA was diluted in 19 µL of bidest. H_2_O. The solution was incubated at 65 °C for 10 min and then for 3 min on ice. The RNA was also transcribed in a reaction volume of 40 µL using the same MLV RT-Mix described for random priming above, containing 100 pmols of target-specific primers. The target-specific primers were: RT-*GUSB*-reverse 5′-CTCGC AAAAG GAACG CTGC-3′; RT-*ABL1*-reverse 5′-CCTCC CTTCG TATCT CAGCG-3′. After adding 21 µL of RT-Mix, the reaction mixture was incubated for 2 h at 37 °C, followed by 5 min at 65 °C, and then stored at −20 °C. For the subsequent qPCR, 3 µL of the final cDNA reaction mix was used per reaction and well. 

### 2.4. cDNA Synthesis Employing Random Hexamer and Single Specific Priming Using SuperScript IV Reverse Transcriptase

The second commercially available RT/cDNA first-strand synthesis kit used was the SuperScript IV reverse transcriptase system (Invitrogen/Thermofisher Scientific, cat. no. 18090010), MLV mutant featuring enhanced fidelity and thermostability and reduced RNase activity. In total, 1 µg of total RNA was transcribed according to the manufacturer’s instructions, using 10 U/µL SS IV RT and 200 ng (=100 pmol) pdN6 random primer (Roche) or 50 pmol target-specific primers. At first, the total RNA and primer/RT-Mix was incubated for 5 min at 65 °C; then, 7 µL of RT reaction mix was added. Afterwards, the reaction mixture was incubated at 52 °C for 10 min and 80 °C for 10 min for specific priming using the target-specific primers RT-*GUSB*-reverse 5′-CTCGC AAAAG GAACG CTGC-3′ and RT-*ABL1*-reverse 5′-CCTCC CTTCG TATCT CAGCG-3′. The reaction mixture containing the random primer hexamers was incubated for 10 min at room temperature. For the subsequent qPCR, 3 µL of the final cDNA reaction mix with the volume of 40 µL was used per reaction and well.

### 2.5. Measurement of GUSB and BCR::ABL1 Transcript Levels by qRT-PCR

The TaqMan qRT-PCR detection system (TaqMan 7500 Fast Real-Time PCR System, TermoFisherScientific/Applied Biosystems, Waltham, MA, USA) was used for the amplification and quantification of *GUSB* control and the e14-a2 type *BCR::ABL1* fusion gene transcript-derived cDNAs, as described previously [8]. In brief, qRT-PCR for *BCR::ABL1* transcripts was performed in triplicate using 96-well plates. Eight serial dilutions of commercially available plasmid pIRMM0099 (ERM-AD623/“Institute for Reference Materials and Measurements”) equivalent to 3, 15, 30, 300, 3000, 30,000, and 300,000 and 3 × 10^6^ copies per reaction served as plasmid standards. The following served as primers for *BCR::ABL1* detection: ENF501sense 5′-TCCGC TGACC ATCAA YAAGG A-3′ and ENR561antisense 5′-CACTC AGACC CTGAG GCTCA A-3′. The expected size of the e14a2 *BCR::ABL1* amplicon is 149 bp. A *BCR::ABL1* specific probe ENP541-MGBsense (6FAM-CCCTT CAGCG GCCAGT-MGB) was used. The *GUSB* specific primers were ENR1162 sense 5′-CCGAG TGAAG ATCCC CTTTT TA-3′ and ENF1102antisense 5′-GAAAA TATGT GGTTG GAGAG CTCAT T-3′. The resulting *GUSB* amplicon is 101 bp in length. For the detection of *GUSB,* the specific probe ENPr1142sense was used (NED-CCAGC ACTCT CGTCG GTGAC TGTTC A-MGB). The specific primer pair for *ABL1* was: ENF 1003 (sense) 5′-TGGAG ATAAC ACTCT AAGCA TAACT AAAGGT-3′ and ENR 1063 (antisense) 5′-GATGT AGTTG CTTGG GACCCA-3′. The PCR fragment is 124 bp long; the *ABL1* specific probe was ABL-1043V-MGB antisense (VIC-CATTT TTGGT TTGGG CTTC-MGB). For the detection of *ABL1*, a combination of the reporter dye VIC and the quencher NFQ-MGB was used. For measuring *BCR::ABL1*, FAM and NFQ-MGB were employed as reporter dye and quencher, respectively. NED served as the reporter dye for *GUSB*, NFQ-MGB was used as the quencher. The final concentrations for all primers and probes were 10 μM per qPCR reaction. Real-time duplex PCR (*BCR::ABL1* and *GUSB* in one single well) was performed in a total volume of 20 μL per reaction using 10 μL of a TaqMan Fast Advances Master Mix (ThermoFisherScientific/Applied Biosystems). The PCR steps were: first, a holding stage at 50 °C for 2 min followed by an additional holding stage at 95 °C for 20 s. Thereafter, 45 cycles followed, comprising 95 °C for 3 s and 60 °C for 45 s. The criteria for passing for each TM run were applied, as described previously [8]. For comparative data evaluation, absolute and relative numbers [%] of detected *GUSB* and *BCR::ABL1* molecules were used for the calculation of the clinically relevant *BCR::ABL1* quotient (X [%] = [number of molecules *BCR::ABL1*]/[number of molecules *GUSB*] × 100), in compliance with the guidelines of Foroni and coworkers [9].

### 2.6. Data Calculation and Diagnostic Outcome

For *BCR::ABL1* routine diagnostics, duplicate qPCR analyses were performed with 3 µL of cDNA reaction mix per qPCR reaction and well (corresponding to a total of 150 ng of RNA in two wells). For the RT experiments described here, triplicates (matching 500 ng of RNA) or hexaplicates (entire 1 µg of sample RNA) were performed, depending on the amount of cDNA available. In the latter case, the entire cDNA reaction volume was distributed over six qPCR reaction wells, and the resulting values from the six measurements were added up so that the total number of transcripts from 1 µg RNA could be determined. When triplicates were performed, the sum of all measurements was also calculated, and the value was extrapolated to a virtual amount of 1 µg RNA for better comparability between the different RT experiments. The same holds true for the Acrometrix™ *BCR::ABL1* reference panel experiments. For the calculation of the absolute transcript numbers of *BCR::ABL1*, *GUSB*, and *ABL1* and the *BCR::ABL1*/*GUSB* or *BCR::ABL1*/*ABL1* quotients, all measured values of the triplicates or hexaplicates of one sample were summed up; no means of triplicate and hexaplicate values were considered [9]. Furthermore, it was only possible to calculate the laboratory quotients Log (*BCR::ABL1*/HKG%) according to Cross et al., 2015 [5]. Log (*BCR::ABL1*/HKG %IS) could not be calculated due to the lack of appropriate conversions factors for MLV specific priming and the SuperScript IV enzyme system (random and specific priming). Although it is possible in principle to establish conversion factors using the Acrometrix panel, this was not accomplished in our experimental setting because, for the comparative results, it seemed not mandatory to us to stick with the quotient [% IS].

## 3. Results

### 3.1. Performance Comparison of MLV-RT and SuperScript IV-RT Using the Acrometrix™ BCR::ABL1 Reference Panel

**Figure 1:**
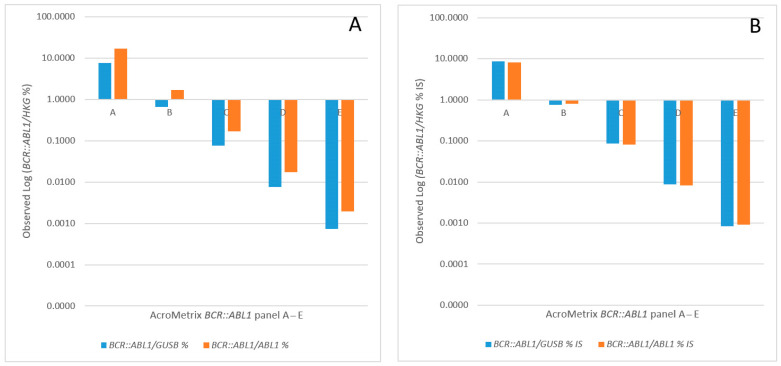
Validation of our routine diagnostic workflow using the Acrometrix™ *BCR::ABL1* panel that consists of 5 vials, here termed A, B, C, D and E. (**A**) TaqMan qRT-PCR employing our standardized reverse transcription protocol (MLV RT combined with random hexamer priming) revealed great linearity across the expected MR range (*BCR::ABL1* 10% down to 0.0032%). (**B**) The laboratory-specific conversion factors 1.147 and 0.476 were used to achieve the acceptable range of IS accuracy when *GUSB* and *ABL1* served as housekeeping genes (HKG), respectively. All data are based on triplicate assays. Abbreviations: HKG, housekeeping gene; IS, international scale. A Log (*BCR::ABL1*/*GUSB* %IS) of 0.1 corresponds to MMR.

We used the Acrometrix™ *BCR::ABL1* panel, the first commercially available cell-based secondary reference panel for *BCR::ABL1* quantification on the IS [25], for the quality assurance and validation of our routine diagnostic workflow, including our actual laboratory-specific conversion factor. Both are based on reverse transcription, employing a combination of MLV RT and random hexamer priming followed by TaqMan qPCR according to a standardized protocol, as described previously [8]. As shown in Figure 1 the Acrometrix™ *BCR::ABL1* panel demonstrates excellent usability for monitoring the clinically relevant *BCR::ABL1* Log reduction points, featuring a dynamic range from 10% (panel A) to 1% (panel B), 0.1% = MMR (panel C), 0.01% = MR4 (panel D), and 0.0032% = MR^4.5^ (panel E). The respective conversion factors (CF) for *GUSB* (1.147) and *ABL1* (0.476) HKG were used to achieve satisfactory IS accuracy (Figure 1B) for both HKG.

**Figure 2:**
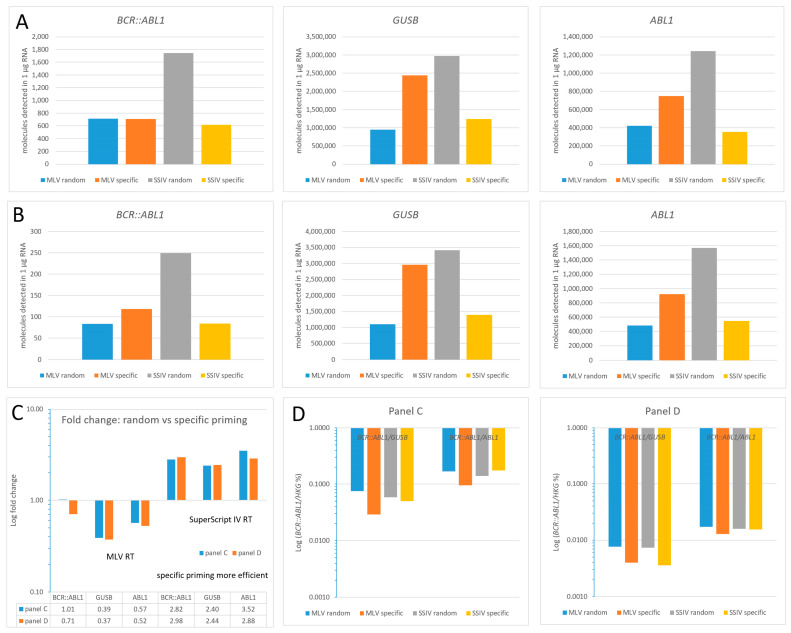
Calculated number of target molecules (*BCR::ABL1*, *GUSB*, and *ABL1*) per 1 µg of RNA detected by MLV-RT and SuperScript IV RT in representative *BCR::ABL1* reference panels (**C**) (0.1% = MMR; see Figure panel (**A**,**D**) (0.01% = MR4; see Figure panel (**B**)). All data are based on the summation, not mean, of triplicate assays, according to the formula X [%] = [total number of molecules *BCR::ABL1*]/[total number of molecules HKG] × 100, in compliance with the guidelines of Foroni et al., 2011 [9]. (**C**) Fold changes (FC) of random vs. specific priming efficacy; Figure panel (**D**): Display of clinically relevant Log (*BCR::ABL1*/HKG%). Laboratory-specific quotients are given since no CF for the use of specific primers is available. Abbreviations: HKG, housekeeping gene; MMR, major molecular response; MR4, molecular response; CF, conversion factor; FC, fold change.

Since, obviously, the Acrometrix™ *BCR::ABL1* panel is an excellent tool for monitoring *BCR::ABL1* assay performance, we used it to comparatively investigate the impact of two different reverse transcriptases (MLV RT vs. SuperScript IV RT) on *BCR::ABL1* assay performance. Furthermore, the influence of the cDNA priming method was analyzed by using either target-specific primers (each one for *GUSB* and *ABL1*) or a mixture of random hexamers, as commonly recommended by the majority of RT manufacturers and by the *BCR::ABL1* secondary reference panel protocol [25]. The total RNA extracted from each of the reference panel vials A-E was equally split into four portions. Four reverse transcription reactions were performed in parallel with either MLV RT (random vs. specific priming) or SuperScript IV RT (each enzyme with random vs. specific priming). The resulting molecule numbers detected by TaqMan qPCR were upcalculated to a 1 µg RNA input for better data comparability. Two representative panels, C (0.1% = MMR) and D (0.01% = MR4), are depicted in Figures 2A and 2B, respectively.

As shown in Figure 2, where the measured copy numbers of *BCR::ABL1*, *GUSB*, and *ABL1* detected by the four experimental settings were comparatively analyzed, we found that the number of targets (low- or high-copy) affects the efficacy of the respective priming type (random vs. specific). Moreover, the type of RT (MLV RT vs. SuperScript IV RT) has a crucial impact. So, when looking at MLV RT only, there was no (A = reference panel C) or only a minor effect (B = reference panel D) in the detection of *BCR::ABL1* low copy numbers (*n* < 800), irrespective of the priming type applied. In contrast, for the high-copy targets, *GUSB*/*ABL1* random priming revealed a 0.39-fold/0.57-fold (reference panel C) and 0.37-fold/0.52-fold (reference panel D) decrease in the number of detected molecules, respectively, when compared to specific priming. Thus, specific priming seems superior when it comes to the measurement of the HKG *GUSB* or *ABL1*, in contrast to the detection of *BCR::ABL1* targets. The respective fold changes are shown in Figure 2C.

However, the high-fidelity RT enzyme SuperScript IV displays completely different reverse transcription characteristics, as, for all targets, random priming turned out to be superior to specific priming, irrespective of the target copy number and reference panel tested. This feature is reflected by the FC (random vs. specific priming) given in Figure 2C, where the FCs for the SuperScript IV RT range between 2.40 and 3.52 for both reference panels. This is in strong contrast to the performance of MLV RT that features FCs between 1.01 and 0.37 (random priming was inferior to specific priming).

What may this mean for the resulting clinical diagnostic outcome? The Log (*BCR::ABL1*/HKG %) is calculated according to the formula X [%] = [total number of molecules *BCR::ABL1*]/[total number of molecules HKG] × 100, in compliance with the guidelines of Foroni et al., 2011 [9]. Therefore, changes in the denominator corresponding to the number of detected HKG targets display a higher impact on the diagnostic outcome than the numerator (i.e., detected *BCR::ABL1* copies). This may lead to a diagnostic bias. As shown in Figure 2A, in the case of MLV-RT (reference panel C), about equal *BCR::ABL1* copies (=numerator) were detected, irrespective of the priming type, while the detection of the corresponding HKG *GUSB* turned out superior when specific priming was used. This yielded lower Log (*BCR::ABL1*/*GUSB*%) values when specific priming in combination with MLV RT was applied for reverse transcription (Figure 2D). The same held true when *ABL1* served as HKG. Thus, paradoxically, assaying clinical samples with MLV RT and specific priming may result in better-responding patients (i.e., lower Log (*BCR::ABL1*/*GUSB*%) values) compared to random priming. In the case of SuperScript IV RT, we found that the overall performance superiority of random priming affects both the numerator and denominator quite equally, and therefore, the cDNA priming type may account for only minor changes in the diagnostic outcome.

### 3.2. Performance Comparison of MLV RT and SuperScript IV RT Using Clinical Samples

**Figure 3:**
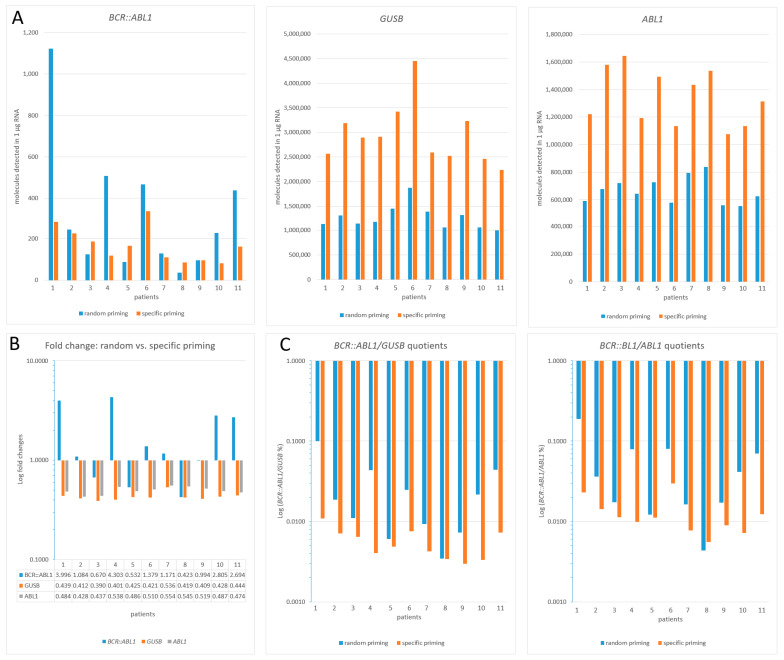
Calculated number of target molecules (*BCR::ABL1*, *GUSB*, and *ABL1*) per 1 µg of RNA detected in clinical routine samples of CML patients (*n* = 11). For reverse transcription, MLV RT and random or specific priming were used (**A**). All data are based on the summation, not mean, of triplicate assays, according to the formula X [%] = [total number of molecules *BCR::ABL1*]/[total number of molecules HKG] × 100, in compliance with the guidelines of Foroni et al., 2011 [9]. (**B**): Fold changes (FC) of random vs. specific priming efficacy; (**C**): Display of clinically relevant Log (*BCR::ABL1*/HKG%) values. Lab-specific quotients are only given for better comparability to Figure 4.

**Figure 4:**
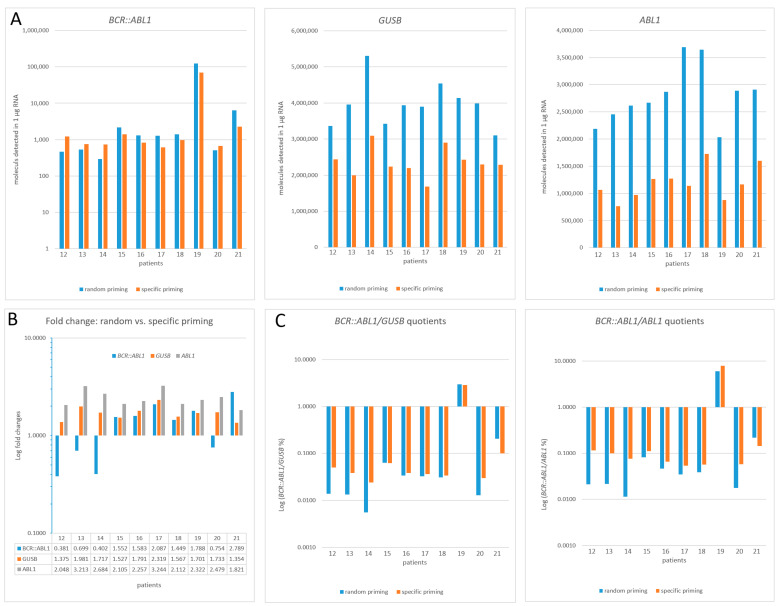
Calculated number of target molecules (*BCR::ABL1*, *GUSB*, and *ABL1*) per 1 µg of RNA detected in clinical routine samples of CML patients (*n* = 10). For reverse transcription, SuperScript IV RT (SSIV) and random or specific priming were used (**A**). All data are based on the summation, not mean, of triplicate assays, according to the formula X [%] = [total number of molecules *BCR::ABL1*]/[total number of molecules HKG] × 100, in compliance with the guidelines of Foroni et al., 2011 [9]. (**B**): Fold changes (FC) of random vs. specific priming efficacy; (**C**): Display of clinically relevant Log (*BCR::ABL1*/HKG%) values. Laboratory-specific quotients are given since no CF for SSIV RT is available.

To verify the Acrometrix™ *BCR::ABL1* reference panel-based findings under routine diagnostic conditions, we performed analogous experiments with clinical specimens derived from routine laboratory *BCR::ABL1* monitoring. Eleven samples (patients 1–11; for clinical data, see Table 1) were assayed using MLV RT in combination with random vs. specific priming (Figure 3A–C). Due to the limitation of the clinical sample material, 10 different clinical RNA specimens (patients 12–21) had to be used for testing the SuperScript IV RT in an analogous manner (Figure 4A–C).

The detection of *BCR::ABL1* targets (Figure 3A, copy range per 1 µg RNA: 12 (patient 8) to 375 (patient 1) when random-primed) after MLV RT-based reverse transcription (random vs. specific priming) revealed that random priming was superior to specific priming if the number of *BCR::ABL1* targets exceeded the number of about 70. This resulted in an overall FC of 1.82 (ratio random/specific priming) for the *BCR::ABL1* target. At target copy numbers below 70, specific priming seems to become more and more superior (Figure 3A, patients 3, 5, 7, 8, 9), indicating a strong reciprocal relationship of target copy numbers with the priming type efficacy, as emphasized in Figure 3B, where the corresponding fold changes (FC: random vs. specific priming) are shown. For both the high-copy-number targets *GUSB* and *ABL1*, the MLV RT in combination with specific priming detected nearly twice as many target molecules when compared to random priming, resulting in a mean FC (ratio of random vs. specific priming) of 0.43 and 0.49 for *GUSB* and *ABL1* targets, respectively (Figure 3B). Since, consequently, MLV RT in combination with specific priming leads to a twice-as-high denominator as with random priming, the resulting Log (*BCR::ABL1*/*GUSB*%) values suggest a better diagnostic outcome than when patients are monitored with random-primed assays (Figure 3C). In the extreme case (patient 1), this accounts for one entire *BCR::ABL1* Log reduction step (random 0.1 = MMR vs. specific 0.0109 = MR^4^). Since at *BCR::ABL1* target copy numbers below 70, random and specific priming seem to generate converging target numbers (Figure 3A, patients 3, 5, 7, 8, 9), the resulting Log (*BCR::ABL1*/*GUSB*%) reduction differences (random vs. specific priming) becomes less and less prominent (mean random 0.0074 (range 0.0034 to 0.0111 vs. mean specific 0.0044 (range 0.0030 to 0.0064)). This is most visible for patient 8, for whom an identical Log (*BCR::ABL1*/*GUSB*%) of 0.0034 could be stated, irrespective of the type of priming. However, except for patient 1, the approximate two-fold differences observed in the Log (*BCR::ABL1*/*GUSB*) are in the acceptable range (up to three-fold) of variability observed when using a conversion factor (CF) [26].

In the case of SuperScript IV RT, we confirmed the overall performance superiority of random priming over specific priming, as already observed for the Acrometrix™ *BCR::ABL1* reference panel (Figure 2). For the low-copy target *BCR::ABL1*, random priming detected about 1.88-fold (mean of patients #15–19, #21) more molecules per 1 µg of RNA compared to specific priming (Figure 4A). At molecule numbers below 250 (patients #12–14, #20), specific priming becomes superior, featuring an FC of 0.56 (mean) for *BCR::ABL1*. For the high-copy targets, random priming, when compared to specific priming, revealed a 1.70-fold and 2.43-fold increase (mean) in the number of detected molecules for GUSB and ABL1, respectively. Thus, except for patients #12–14 and #20, the absolute assay sensitivity (per 1 µg RNA) seems to benefit from the combination of SSIV RT and random priming (Figure 4B). This affects both the numerator and denominator quite equally, and therefore, despite a weak trend towards random priming superiority, the cDNA priming type accounts only for minor changes in the diagnostic outcome, which are within the acceptable range (up to threefold) of variability [26] (Figure 4C). However, for clinical samples with *BCR::ABL1* target numbers below 250 (patients #12–14, #20), a 3.1-fold (mean random: 0.0114 vs. mean specific: 0.0353) Log (*BCR::ABL1/GUSB%*) reduction was observed. This points to a general trend where the corresponding patients seem to respond better when assayed with specific priming (i.e., lower Log (*BCR::ABL1/GUSB%*) values) than when specific priming was applied in combination with SuperScript IV (Figure 4C).

### 3.3. SuperScript IV in Combination with Specific Priming Grants the Highest Sensitivity for BCR::ABL1 Detection at Target Copy Numbers <50

**Figure 5:**
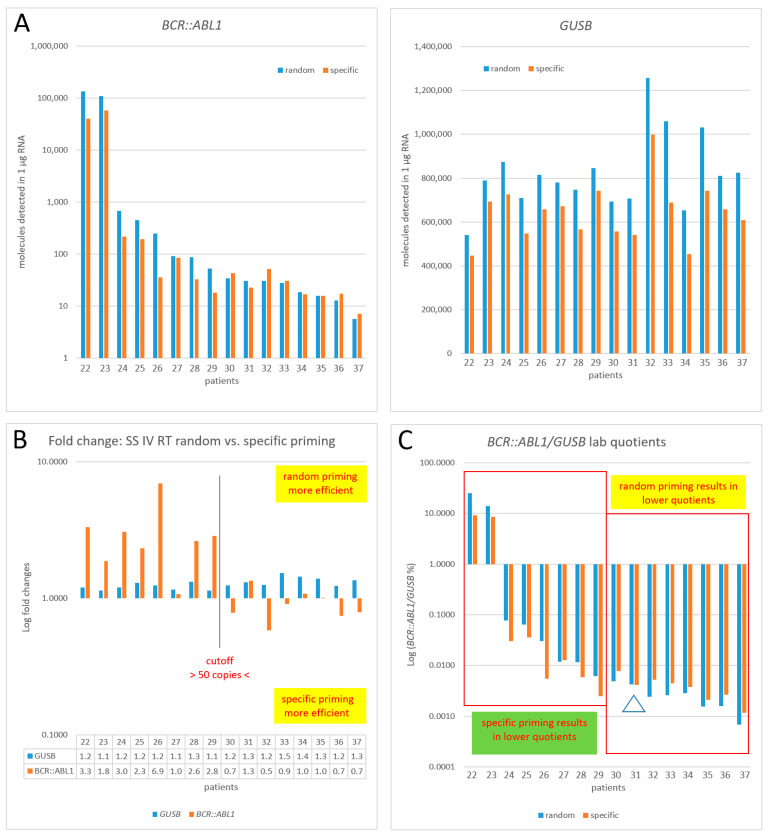
Number of target molecules (*BCR::ABL1* and *GUSB*) per 1 µg of RNA detected in clinical routine samples of CML patients (*n* = 16). Patient samples are ordered according to decreasing *BCR::ABL1* numbers (when random priming was applied). For reverse transcription, SuperScript IV RT and random vs. specific priming were used (**A**). All data are based on the summation, not mean, of hexaplicate assays, according to the formula X [%] = [total number of molecules *BCR::ABL1*]/[total number of molecules *GUSB*]*100, in compliance with the guidelines of Foroni et al., 2011 [9]. (**B**): Fold changes (FC) of random vs. specific priming efficacy; (**C**): Display of clinically relevant Log (*BCR::ABL1*/*GUSB*%). Lab-specific quotients are given since no valid CF for SSIV RT is available. The open triangle denotes an outlier.

In order to identify the best-performing reverse transcription procedure for monitoring therapy-free remission (TFR) and patients before stopping TKI treatment decisions, we performed analogous experiments with clinical specimens (*n* = 16) that are representative for the types of samples regularly encountered by a routine diagnostic workflow. Sixteen samples (patients #22–#37; for clinical data, see Table 1) were assayed using SuperScript IV (SSIV) in combination with random vs. specific priming (Figure 5A–C). *BCR::ABL1* copy numbers, as shown in Figure 5A in decreasing order, ranged between 135,000 (pat #22) and 6 (pat #37) when random priming was applied. According to our lab-specific workflow, only *GUSB* was employed as HKG (simultaneously tested with *BCR::ABL1* within the same well) in this experiment. This enabled the execution of hexaplicate assays so that the complete amount of cDNA corresponding to 1 µg of the sample RNA was used up.

While for *GUSB*, a similar superiority of random over specific priming was detected (mean FC of 1.28, SD 0.011) as in the former experiment in Figure 4, an analysis of *BCR::ABL1* confirmed our former finding that, below a certain copy number cut-off (here, *n* = 50), specific priming becomes superior to random priming for *BCR::ABL1*. This is depicted in Figure 5B, where patients #22 to #29 (*n* = 8) show random priming superiority (mean FC of 3.02, SD 1.64), while at copy numbers <50 (patients #30 to #37), specific priming became more efficient (mean FC 0.90, SD 0.22). For *GUSB*, random priming always shows a higher efficacy in terms of target molecule detection (Figure 5B, blue columns). Within the range of the cut-off, two outliers (patient #27 and patient #31, depicted by open triangles) suggest that the exact cut-off may be slightly floating depending on experimental conditions. Thus, in the former experiment in Figure 4, the cut-off was at a *BCR::ABL1* copy number <250, where specific priming outnumbered random priming (compare Figure 4B).

These findings find expression in the diagnostic important Log (*BCR-ABL1*/*GUSB*%) level quotient that clearly demonstrates that, in this experiment, the numerator (*BCR::ABL1*) and denominator (*GUSB*) follow different kinetics in reverse transcription depending on the priming type. At *BCR::ABL1*, copy numbers below the cut-off random priming result in lower Log (*BCR::ABL1*/*GUSB*%) quotients, making patients appear better-responding (mean FC 0.67, SD 0.16, range 0.46–1.02), while at copy numbers above the cut-off, an opposite trend could be observed (mean FC 2.48, SD 1.4, range 0.92–5.62). Although the acceptable range of variability observed when using a CF has been described approximately three-fold [26], the absolute number of *BCR::ABL1* molecules detectable in a clinical specimen (per 1 µg RNA) may serve as an excellent basis for clinical decision making.

### 3.4. Optimization of Reverse Transcription Proves Advantageous in Terms of TFR Monitoring

In order to prove the potential benefit of the best possible RT efficacy in terms of *BCR::ABL1* detection, we have compared the diagnostic routine laboratory data ((Log BCR-ABL/*GUSB* %IS) shown in Table 1) of 26 patients (patient ID: 12 to 37) to an optimized RT protocol. In contrast to the routinely used method (MLV RT, random priming), this protocol combined SuperScript IV RT with random or specific priming. Quantitative PCR was performed on a 1 µg RNA/cDNA equivalent instead of 0.150 µg (1/6 of 1 µg RNA) only, the amount that applies for regular *BCR::ABL1* routine qRT-PCR assaying (for details, see Materials & Methods). As shown in Table 2, the resulting higher sensitivity led to 12 individual Log level changes each for random and specific priming. For random priming, six patients (#14, #29–32, #36) stepped up one Log level (yellow), and five patients (#12, #13, #26–28) stepped up two Log levels (orange). One patient (#37) stepped down one Log level (MR^4.5^ -> MR^5^). Specific priming performed quite similar (stepped up one Log level, *n* = 7; two Log levels, *n* = 4). One patient (#21) reached MMR, although no MMR was diagnosed in routine diagnostics. Obviously, these Log level shifts were due to a higher cDNA amount used in general but also to the priming type-dependent RT reaction kinetics differing for low- (*BCR::ABL1*) and high-copy targets (*GUSB*). For example, in the case of patient #32 (compare Figure 5B), the fold changes (random vs. specific priming) for the reverse transcription of *GUSB* (FC = 1.26) differ considerably from those of *BCR::ABL1* (FC = 0.58). This concurs with the “better” diagnostic outcome (patient #32 in Table 2, MR^4.5^) when random priming was applied compared to specific priming. Specific priming increased the number of detected *BCR::ABL1* molecules, resulting in a Log level shift MR^4.5^ -> MR^4^. This is an excellent example of how alternative priming can impact the Log level calculation, making the patient’s residual tumor load appear more or less eminent.

## 4. Discussion

In diagnostic applications, it is assumed that RT enzymes perform a stoichiometrically correct reverse transcription. Low- (*BCR::ABL1*) and high-copy-number (*GUSB* or *ABL1*) targets should be proportionally processed with identical efficacy within a defined range of sample RNA concentrations. In particular, this is of great importance in CML routine *BCR::ABL1* monitoring, as the number of *BCR::ABL1* cDNA targets is considered to exactly reflect the tumor load.

Our experiments with MLV RT and SuperScript IV on the Acrometrix™ *BCR::ABL1* reference panel and 37 clinical specimens revealed that the question regarding the desired 1:1 stoichiometry has to be discussed, as already addressed by the findings of others [11,13,19,20,21,22]. We found that the RT type (MLV vs. SuperScript IV), type of priming (random vs. specific), and target copy number (> or <50 copies of *BCR::ABL1*) are major factors for diagnostic data variation, and it became clear that the influence of RT on the diagnostic outcome has been clearly underestimated so far.

The key difference between both tested RT enzymes was their divergent processivity of high- and low-copy RNA targets with respect to the priming type. When the *BCR::ABL1* RNA target copy number fell below a distinct threshold (<200 in Figure 4; <50 in Figure 3 and Figure 5), specific priming tended to become superior over random priming. Obviously, the kinetics of both enzymes harmonize when very-low-copy targets are present in a high background of human total RNA. Thus, specific priming can be desirable when it comes to the detection of very few *BCR::ABL1* targets, e.g., in the case of TFR monitoring or therapy decisions regarding the participation of CML patients in TKI cessation studies, as suggested previously [13].

The different properties of the two enzymes MLV and SuperScript IV may best be explained by comparing the respective working protocols given by the manufacturers. While MLV RT-driven cDNA synthesis takes place at 37 °C for 2 h, SuperScript IV RT performs at 52 °C for 10 min, pointing to a remarkable temperature resistance (steady ternary structure of polypeptide) combined with a much higher processivity according to the temperature coefficient Q10 rule given by the van’t Hoff equation, saying that the reaction rate can double for each 10 °C rise in temperature. Recombinant RTs that can work at elevated reaction temperatures were found to be superior to low-temperature-processing enzymes and have been recommended for low-RNA-input applications [27]. Irrespective of the applied priming type, one should expect nearly identical results for the same RNA sample and RT enzyme. However, our data showed that the reverse transcription of high- and low-copy-number target RNA is divergent depending on the priming type. Since the RNA target numbers in all reactions were identical, there must have been more successful *BCR::ABL1*-specific initiations in the random primed samples than in the specific primed sample. For specific priming, just one initiation per target molecule may be expected theoretically. It seems that random priming can lead to multiple initiations on one single RNA target that results in multiple cDNA molecules per RNA template molecule. This mechanism is supported by SD synthesis activity, known to be a natural feature of RT enzymes essential for retroviral replication in vivo. The SD synthesis activity of SuperScript IV in combination with random priming under high reaction temperatures may favor multiple initiations per RNA template, entailing some sort of linear “pre-amplification” of targets during cDNA synthesis. As a consequence of this RT side activity, it leads to an overestimation of themeasured targets in qPCR. It cannot be excluded that specific primers also contribute to SD synthesis activity by the unspecific binding of their 3′ ends in a less random manner.

Moreover, primer binding kinetics are highly dependent on the reaction temperature. At 37 °C, random primer target binding may be comparable to specific primer binding, whereas at 52 °C, random priming can be expected to be much less efficient (Tm value and Gibbs free energy (ΔG)). Thus, during cDNA initiation with MLV RT, the abundancy of template-bound random hexamers (200 ng = 100 pmol) may outnumber the number of functional RT molecules and lead to a general underrepresentation of processed RNA targets (RT is a limiting factor). Of course, the amount of the primer and enzyme, i.e., the relation of the primer and enzyme, could be optimized with regard to the expected number of respective templates. The impairment of MLV RT and SuperScript IV RT in combination with specific or random priming may not be reflected by the resulting diagnostically relevant Log (*BCR::ABL1*/HKG%) value, since a small numerator (*BCR::ABL1*) can be easily compromised by changes in the denominator (*GUSB* or *ABL1*). Patients with small *BCR::ABL1* target numbers show deeper molecular responses (DMR) when assayed with random priming (i.e., lower Log (*BCR::ABL1*/*GUSB*%) values) than when specific priming was applied in combination with SuperScript IV (Figure 4C), even if the number of detected *BCR::ABL1* molecules was higher with random-primed cDNA (Figure 4A, patients #15–#19). As a consequence, if an optimized protocol is applied (SuperScript IV combined with a random hexamer or specific priming depending on the *BCR::ABL1* target copy number> or <50, respectively), the resulting higher sensitivity can lead to individual Log level changes. Alternative priming may have an impact on the Log level calculation, making the patient’s residual tumor load appear more or less eminent. In other words, the goal must not be to have the highest amount of the housekeeping gene but to have the correct relation of the housekeeping gene and target gene. In our regular routine monitoring, this may be of minor importance, as a higher sensitivity of a lab-specific method will be corrected by the proper CF factor. The limits of variation can be up to three-fold when using a CF [26].

The sensitivity of a diagnostic laboratory is always normalized to that of the corresponding reference laboratory or its given standards—here, the Acrometrix™ *BCR::ABL1* reference panel that was also established using random priming combined with the ABI High-Capacity cDNA reverse-transcription kit (ThermoFisher Scientific, order no. 4368814; [25]). However, when it comes to TFR monitoring or TKI discontinuation decisions, an increase in assayed RNA amounts and the use of distinct RT/priming combinations may be advantageous for achieving the highest *BCR::ABL1* assay sensitivity possible. For us, it seems advantageous to measure *BCR::ABL1* copy numbers additionally in a distinct amount of RNA (e.g., 1 µg), thereby omitting the influence of denominators (HKG) varying with the RT and priming type. This approach would require the exact determination of the RNA quantity and quality, the latter to be analyzed by chip-based methods calculating RNA integrity numbers (e.g., RIN calculated by an Agilent 2100 Bioanalyzer System). Since the detection of single or sets of housekeeping genes also depends on the global RNA quality, more efficient and effective methods are imaginable, such as looking at the total levels of gene expression across all genes, as suggested by others [28].

The ELN panel agrees that TFR is the significant goal in CML management and that TKI treatment discontinuation should be considered in patients with durable DMR [2,29,30,31]. Multiple cohort studies imply relapse (loss of MMR) in about 50% of patients after TKI cessation, regardless of the TKI used [32,33]. Most molecular recurrences occur within the first 6–8 months after TKI discontinuation and trigger the restart of TKI therapy [30]. Since the duration of TKI therapy and DMR are considered to be the most important prognostic factors [3,34] for TFR success, the application of the *BCR::ABL1* monitoring method with the highest sensitivity is crucial. Our patients (Table 2, #12, #13, #30, #31), after TKI discontinuation (MR^4.5^), stepped up one or two Log BCR-ABL/HKG % IS values when a more sensitive method was applied in monitoring after TKI discontinuation. Since, for these patients of our cohort, the real tumor load may be higher than that monitored by routine *BCR::ABL1* assays (MR^4.5^), future follow-up may be informative. On the record, all four patients are still in TFR.

## 5. Conclusions

The employment of modern, convenient-to-use cDNA first-strand synthesis kits has led to a common disregard for the influence of cDNA synthesis in molecular methods. Varying efficacies of different RT enzyme grades for processing high- and low-copy-number RNA targets depend on the priming type and can introduce a bias into the resulting gene expression data, shifting Log *BCR::ABL1* % IS values and potentially impacting the monitoring outcome if the proper CF will not be applied. In the case of *BCR::ABL1* monitoring for TKI discontinuation, we consider *BCR::ABL1* assays with the best possible sensitivity crucial but also underline the importance of inter-laboratory harmonization and the permanent validation of laboratory methods on the basis of a series of highly standardized reference samples (i.e., the Acrometrix™ *BCR::ABL1* reference panel). However, for inter-laboratory harmonization, the proper conversion factor according to the CML international standard (IS) has to be reevaluated, especially if the RT type or cDNA priming is changed.

## Figures and Tables

**Table 1:** CML patients analyzed by routine/experimental diagnostics (*BCR::ABL1* qRT-PCR).

Patient	Age [Years]	Sex	*BCR::ABL1* Breakpoint	Therapy *	LogLevel [%IS] **
1	36	f	e13a2	no therapy	MR^4^
2	49	f	e14a2	Dasatinib	MR^4^
3	65	m	e14a2	Dasatinib	MR^4^
4	64	m	e14a2	Nilotinib	MR^4^
5	22	f	e13a2	Bosutinib	MR^4^
6	70	f	e14a2	Asciminib	MMR
7	75	m	e13a2, e14a2	no therapy	MR^4.5^
8	80	m	e14a2	Nilotinib	MR^4.5^
9	75	m	e14a2	no therapy	MR^4.5^
10	74	f	e13a2, e14a2	Imatinib	MR^4.5^
11	62	f	e13a2	Bosutinib	MR^4.5^
12	64	f	e13a2	no therapy	MR^4.5^
13	56	m	e14a2	no therapy	MR^4.5^
14	80	f	e14a2	Imatinib	MR^4.5^
15	43	f	e13a2	Nilotinib + Asciminib	MMR
16	52	m	e13a2	Bosutinib	MMR
17	62	m	e13a2	Imatinib	MMR
18	60	m	e13a2	Bosutinib	MMR
19	56	m	e13a2	Dasatinib	no MMR
20	56	f	e13a2	Ponatinib	MMR
21	35	m	e14a2	Nilotinib	no MMR
22	37	m	e14a2	Nilotinib	no MMR
23	63	f	e14a2	Asciminib	no MMR
24	39	m	e13a2, e14a2	Nilotinib	MMR
25	65	f	e13a2	Imatinib	MMR
26	85	m	e13a2, e14a2	Imatinib	MR^4.5^
27	63	f	e14a2	Dasatinib	MR^4.5^
28	64	f	e13a2	Dasatinib	MR^4^
29	54	m	e13a2, e14a2	Bosutinib	MR^4.5^
30	30	f	e14a2	no therapy	MR^4.5^
31	81	f	e13a2, e14a2	no therapy	MR^4.5^
32	72	m	e14a2	Dasatinib	MR^5^
33	34	f	e14a2	Nilotinib + Asciminib	MR^4.5^
34	44	f	e13a2, e14a2	Dasatinib	MR^4.5^
35	79	m	e13a2	Nilotinib	MR^4.5^
36	59	f	e13a2, e14a2	Bosutinib	MR^5^
37	41	f	e14a2	Nilotinib	MR^4.5^

* at time of PB sampling. ** data derived from laboratory routine diagnostics (MLV RT, random priming). Abbreviations: m, male; f, female.

**Table 2:** Log (*BCR::ABL1*/*GUSB*%) level changes when comparing data derived from routine laboratory diagnostics (MLV RT, random priming) to corresponding SS IV RT experiments (random/specific priming) from the same clinical sample, as depicted in Figure 4 (*n* = 10, patient IDs: 12–21) and Figure 5 (*n* = 16, patient IDs: 22–37).

Patient ID	Figure No.	Current Therapy	Log Level [%IS] Routine Lab (MLV, Random 0.15 µg RNA)	Log Level (Lab Quotient) SS IV Random 1 µg RNA	Log Level (Lab Quotient) SS IV Specific 1 µg RNA
12	4	no therapy	MR^4.5^	MMR	MMR
13	4	no therapy	MR^4.5^	MMR	MMR
14	4	Imatinib	MR^4.5^	MR^4^	MMR
15	4	Nilotinib + Asciminib	MMR	MMR	MMR
16	4	Bosutinib	MMR	MMR	MMR
17	4	Imatinib	MMR	MMR	MMR
18	4	Bosutinib	MMR	MMR	MMR
19	4	Dasatinib	no MMR	no MMR	no MMR
20	4	Ponatinib	MMR	MMR	MMR
21	4	Nilotinib	no MMR	no MMR	MMR
22	5	Nilotinib	no MMR	no MMR	no MMR
23	5	Asciminib	no MMR	no MMR	no MMR
24	5	Nilotinib	MMR	MMR	MMR
25	5	Imatinib	MMR	MMR	MMR
26	5	Imatinib	MR^4.5^	MMR	MR^4^
27	5	Dasatinib	MR^4.5^	MMR	MMR
28	5	Dasatinib	MR^4^	MMR	MR^4^
29	5	Bosutinib	MR^4.5^	MR^4^	MR^4.5^
30	5	no therapy	MR^4.5^	MR^4^	MR^4^
31	5	no therapy	MR^4.5^	MR^4^	MR^4^
32	5	Dasatinib	MR^5^	MR^4.5^	MR^4^
33	5	Nilotinib + Asciminib	MR^4.5^	MR^4.5^	MR^4^
34	5	Dasatinib	MR^4.5^	MR^4.5^	MR^4^
35	5	Nilotinib	MR^4.5^	MR^4.5^	MR^4.5^
36	5	Bosutinib	MR^5^	MR^4.5^	MR^4.5^
37	5	Nilotinib	MR^4.5^	MR^5^	MR^4.5^

Color code: green, no change in Log level; yellow, loss of one Log level; orange, loss of two Log levels; no color, gain of one Log level. Patients #12, #13, #30, and #31 are still in TFR after TKI discontinuation. TFR, therapy-free remission.

## Data Availability

All data generated in this study have been included in the published article.

## References

[1] Cumbo C., Anelli L., Specchia G., Albano F. (2020). Monitoring of Minimal Residual Disease (MRD) in Chronic Myeloid Leukemia: Recent Advances. Cancer Manag. Res..

[2] Hochhaus A., Baccarani M., Silver R.T., Schiffer C., Apperley J.F., Cervantes F., Clark R.E., Cortes J.E., Deininger M.W., Guilhot F. (2020). European LeukemiaNet 2020 recommendations for treating chronic myeloid leukemia. Leukemia.

[3] Branford S., Yeung D.T., Parker W.T., Roberts N.D., Purins L., Braley J.A., Altamura H.K., Yeoman A.L., Georgievski J., Jamison B.A. (2014). Prognosis for patients with CML and >10% BCR-*ABL1* after 3 months of imatinib depends on the rate of BCR-*ABL1* decline. Blood.

[4] Hanfstein B., Lauseker M., Hehlmann R., Saussele S., Erben P., Dietz C., Fabarius A., Proetel U., Schnittger S., Haferlach C. (2014). Distinct characteristics of e13a2 versus e14a2 BCR-*ABL1* driven chronic myeloid leukemia under first-line therapy with imatinib. Haematologica.

[5] Cross N.C., Hochhaus A., Muller M.C. (2015). Molecular monitoring of chronic myeloid leukemia: Principles and interlaboratory standardization. Ann. Hematol..

[6] Jovanovski A., Petiti J., Giugliano E., Gottardi E.M., Saglio G., Cilloni D., Fava C. (2020). Standardization of BCR-*ABL1* p210 Monitoring: From Nested to Digital PCR. Cancers.

[7] Soverini S., Bassan R., Lion T. (2019). Treatment and monitoring of Philadelphia chromosome-positive leukemia patients: Recent advances and remaining challenges. J. Hematol. Oncol..

[8] Spiess B., Rinaldetti S., Naumann N., Galuschek N., Kossak-Roth U., Wuchter P., Tarnopolscaia I., Rose D., Voskanyan A., Fabarius A. (2019). Diagnostic performance of the molecular BCR-*ABL1* monitoring system may impact on inclusion of CML patients in stopping trials. PLoS ONE.

[9] Foroni L., Wilson G., Gerrard G., Mason J., Grimwade D., White H.E., de Castro D.G., Austin S., Awan A., Burt E. (2011). Guidelines for the measurement of BCR-*ABL1* transcripts in chronic myeloid leukaemia. Br. J. Haematol..

[10] Spiess B., Naumann N., Galuschek N., Rinaldetti S., Kossak-Roth U., Tarnopolscaia I., Felde E., Fabarius A., Hofmann W.K., Saußele S. (2018). The benefit of quality control charts (QCC) for routine quantitative BCR-*ABL1* monitoring in chronic myeloid leukemia. PLoS ONE.

[11] Stanoszek L.M., Crawford E.L., Blomquist T.M., Warns J.A., Willey P.F., Willey J.C. (2013). Quality control methods for optimal BCR-*ABL1* clinical testing in human whole blood samples. J. Mol. Diagn..

[12] Roy S.W., Irimia M. (2008). When good transcripts go bad: Artifactual RT-PCR ‘splicing’ and genome analysis. BioEssays.

[13] Jeromin S., Eder C., Haferlach C., Haferlach T., Kern W. (2019). Impact of assay procedures on detection of MR(4.5) status in chronic myeloid leukemia: Optimization of cDNA synthesis. Int. J. Lab. Hematol..

[14] Menéndez-Arias L., Andino R. (2017). Viral polymerases. Virus Res..

[15] Whiting S.H., Champoux J.J. (1994). Strand displacement synthesis capability of Moloney murine leukemia virus reverse transcriptase. J. Virol..

[16] Martín-Alonso S., Kang D., Martínez Del Río J., Luczkowiak J., Frutos-Beltrán E., Zhang L., Cheng X., Liu X., Zhan P., Menéndez-Arias L. (2022). Novel RNase H Inhibitors Blocking RNA-directed Strand Displacement DNA Synthesis by HIV-1 Reverse Transcriptase. J. Mol. Biol..

[17] Mitra S.W., Goff S., Gilboa E., Baltimore D. (1979). Synthesis of a 600-nucleotide-long plus-strand DNA by virions of Moloney murine leukemia virus. Proc. Natl. Acad. Sci. USA.

[18] Shinnick T.M., Lerner R.A., Sutcliffe J.G. (1981). Nucleotide sequence of Moloney murine leukaemia virus. Nature.

[19] Zhang J., Byrne C.D. (1999). Differential priming of RNA templates during cDNA synthesis markedly affects both accuracy and reproducibility of quantitative competitive reverse-transcriptase PCR. Biochem. J..

[20] Nardon E., Donada M., Bonin S., Dotti I., Stanta G. (2009). Higher random oligo concentration improves reverse transcription yield of cDNA from bioptic tissues and quantitative RT-PCR reliability. Exp. Mol. Pathol..

[21] Stangegaard M., Dufva I.H., Dufva M. (2006). Reverse transcription using random pentadecamer primers increases yield and quality of resulting cDNA. BioTechniques.

[22] Lekanne Deprez R.H., Fijnvandraat A.C., Ruijter J.M., Moorman A.F. (2002). Sensitivity and accuracy of quantitative real-time polymerase chain reaction using SYBR green I depends on cDNA synthesis conditions. Anal. Biochem..

[23] Martín-Alonso S., Frutos-Beltrán E., Menéndez-Arias L. (2021). Reverse Transcriptase: From Transcriptomics to Genome Editing. Trends Biotechnol..

[24] Hughes T., Deininger M., Hochhaus A., Branford S., Radich J., Kaeda J., Baccarani M., Cortes J., Cross N.C., Druker B.J. (2006). Monitoring CML patients responding to treatment with tyrosine kinase inhibitors: Review and recommendations for harmonizing current methodology for detecting BCR-ABL transcripts and kinase domain mutations and for expressing results. Blood.

[25] Cross N.C., White H.E., Ernst T., Welden L., Dietz C., Saglio G., Mahon F.X., Wong C.C., Zheng D., Wong S. (2016). Development and evaluation of a secondary reference panel for BCR-*ABL1* quantification on the International Scale. Leukemia.

[26] Müller M.C., Cross N.C., Erben P., Schenk T., Hanfstein B., Ernst T., Hehlmann R., Branford S., Saglio G., Hochhaus A. (2009). Harmonization of molecular monitoring of CML therapy in Europe. Leukemia.

[27] Zucha D., Androvic P., Kubista M., Valihrach L. (2020). Performance Comparison of Reverse Transcriptases for Single-Cell Studies. Clin. Chem..

[28] Liu X., Li N., Liu S., Wang J., Zhang N., Zheng X., Leung K.-S., Cheng L. (2019). Normalization Methods for the Analysis of Unbalanced Transcriptome Data: A Review. Front. Bioeng. Biotechnol..

[29] Hughes T.P., Ross D.M. (2016). Moving treatment-free remission into mainstream clinical practice in CML. Blood.

[30] Saussele S., Richter J., Hochhaus A., Mahon F.X. (2016). The concept of treatment-free remission in chronic myeloid leukemia. Leukemia.

[31] Hochhaus A., Ernst T. (2021). TKI discontinuation in CML: How do we make more patients eligible? How do we increase the chances of a successful treatment-free remission? Hematology. American Society of Hematology. Educ. Program.

[32] Richter J., Lübking A., Söderlund S., Lotfi K., Markevärn B., Själander A., Stenke L., Deneberg S., Ahlstrand E., Myhr-Eriksson K. (2021). Molecular status 36 months after TKI discontinuation in CML is highly predictive for subsequent loss of MMR-final report from AFTER-SKI. Leukemia.

[33] Saussele S., Hehlmann R., Fabarius A., Jeromin S., Proetel U., Rinaldetti S., Kohlbrenner K., Einsele H., Falge C., Kanz L. (2018). Defining therapy goals for major molecular remission in chronic myeloid leukemia: Results of the randomized CML Study IV. Leukemia.

[34] Shanmuganathan N., Pagani I.S., Ross D.M., Park S., Yong A.S.M., Braley J.A., Altamura H.K., Hiwase D.K., Yeung D.T., Kim D.W. (2021). Early BCR-*ABL1* kinetics are predictive of subsequent achievement of treatment-free remission in chronic myeloid leukemia. Blood.

